# A sulfate-arsenical-ferruginous water affects apoptosis, oxidative stress and the gene expression of inflammatory mediators and of a panel of MicroRNA in IL-1β stimulated human osteoarthritic chondrocytes

**DOI:** 10.3389/fmed.2026.1800406

**Published:** 2026-04-20

**Authors:** Antonella Fioravanti, Patrizia Manica, Iole Seccafico, Giulia Collodel, Elena Moretti, Serafino Carta, Sara Cheleschi

**Affiliations:** 1 Independent Researcher, Siena, Italy; 2 Health Resort of Levico and Vetriolo, Levico Terme, Trento, Italy; 3 Rheumatology Unit, Department of Medicine, Surgery and Neuroscience, Azienda Ospedaliera Universitaria Senese, Policlinico Le Scotte, Siena, Italy; 4 Department of Molecular and Developmental Medicine, University of Siena, Siena, Italy; 5 Section of Orthopedics and Traumatology, Department of Medicine, Surgery and Neurosciences, University of Siena, Policlinico Le Scotte, Siena, Italy

**Keywords:** apoptosis, balneotherapy, chondrocytes, cytokines, microRNA, osteoarthritis, oxidative stress

## Abstract

**Background:**

The beneficial effects of balneotherapy in osteoarthritis (OA) using the sulfate-arsenicalferruginous Levico water (LW) have been recognized by several clinical studies. However, the specific biological properties of LW are partially understood.

**Objective:**

The present study aimed at evaluating the ability of LW in the regulation of apoptosis, oxidative stress and of the gene expression of a panel of articular cartilage markers and MicroRNA (*miRNA*) in human osteoarthritic chondrocytes exposed to interleukin (*IL*)-*1*β.

**Methods:**

Chondrocytes were obtained from the femoral heads of patients with OA who underwent surgery for total hip prostheses. The cells were incubated for 24 h and 48 h with different concentrations of LW, alone or in combination with *IL-1*β (10 ng/ml). Apoptosis and mitochondrial superoxide anion production were detected by cytometry. Gene levels of antioxidant enzymes, B-cell lymphoma (*BCL)2*, cytokines [*IL-1*β*, IL-6*, tumor necrosis factor (*TNF*)-α], metalloproteinases (*MMPs*)1 and 13, type II collagen (*Col2a1*), *aggrecan*, and of a pattern of *miRNA* were evaluated by Quantitative Real-Time PCR analysis (PCR). The involvement of nuclear factor (*NF*)*-*κ*B* was investigated by PCR and immunofluorescence and by pre-incubation with a specific inhibitor (*BAY 117082, IKK*α*/*β).

**Results:**

LW at 50% or 25% concentration counteracted the negative effects of *IL-1*β on cell viability, apoptosis and mitochondrial superoxide anion production and on the gene expression of antioxidant enzymes, in a dose-dependent manner. The treatment of chondrocytes with 50% of LW significantly inhibited the gene expression of pro-inflammatory cytokines, *MMPs* and of *miR-34a* and *miR-181a*, while it upregulated *miR-140* in *IL-1*β stimulated cells. The pre-incubation with *BAY 11–7082, IKK*α*/*β reduced the damage induced by *IL-1*β, and, interestingly, potentiated the properties of LW.

**Conclusions:**

Our data demonstrated the ability of LW to regulate apoptosis, oxidative stress and gene expression of main factors implicated in OA pathogenesis through a possible modulation of *NF-*κ*B* signaling pathway. This study supports the use of balneotherapy with a sulfate-arsenical-ferruginous mineral water in the treatment of OA.

## 1 Introduction

Balneotherapy is an effective non pharmacological, complementary approach commonly used for the treatment of several musculoskeletal disorders (1). Sulfate-arsenical-ferruginous Levico water (LW) (Levico Terme, Italy) is a cold mineral water, characterized by a high fixed residue. It contains a high concentration of sulfate, arsenic, iron, and significant amounts of calcium and magnesium. Its efficacy has been extensively studied in various musculoskeletal conditions (2–6). In osteoarthritis (OA), a beneficial effect on pain, function, quality of life, and a significant reduction in symptomatic drug consumption (nonsteroidal anti-inflammatory drugs, acetaminophen) was observed after a cycle of mud-bath treatment with LW (2, 4, 5). The effects persisted for up to 3 months posttreatment (2). Based on these observations, we hypothesized that the mechanism of action of LW is not limited to mechanical and thermal stimuli but also involves the specific activities of its organic, inorganic, and microbiological components (7).

*In vitro* experiments are a useful and valid approach for studying the properties of mineral water and elucidating its specific biological activities. Various cell models may be used to examine the effects of mineral water as a whole or of singular mineral, organic, or microbial elements (7–9). Most studies have evaluated the specific properties of its mineral elements, such as hydrogen sulfide (10–13), while only a few have tested mineral water as a whole (14–20). The analysis of a single compound may not be sufficient to identify the biological mechanisms responsible for the activity of mineral water, considering the potential synergistic and mutual effects of a mixture containing several oligoelements that characterize each water (7–9).

OA is the most common chronic musculoskeletal disorder and a leading cause of disability worldwide (21). It is characterized by articular cartilage degeneration, subchondral bone sclerosis, and synovial membrane inflammation (22). Multiple factors are involved in its complex pathogenesis, such as proinflammatory cytokines and chemokines, catabolic enzymes [matrix metalloproteinases (*MMPs*)], reactive oxygen species (*ROS*), and nitric oxide (*NO*) (23). A close relationship between the excessive apoptosis of chondrocytes and cartilage destruction in OA has been reported, particularly at its late stage (24). A role for noncoding RNAs, such as microRNA (*miRNA*), has also been demonstrated in the pathophysiology of the disease (25–28).

Previously, we described the beneficial effects of Vetriolo water (Vetriolo Terme, Italy), a strongly acidic sulfate, rich in calcium, magnesium and iron thermal water, in human OA chondrocytes stimulated with interleukin *(IL)-1*β. In this experience, we observed enhanced viability, reduced apoptosis, *NO* release, and inducible nitric oxide synthase (*iNOS*) expression in cells exposed to culture medium prepared with different concentrations of Vetriolo water, suggesting its chondroprotective role (17).

In a recent clinical trial of patients with gonarthrosis, we observed a reduction in the expression of a panel of *miRNA* upregulated in OA, at the end of a cycle of mud-bath therapy (29). We hypothesized that the changes in the *miRNA* profile may be related to hot stimulus and hydrostatic pressure considering that these *miRNA* are known to be temperature- and mechano-responsive (30–32).

Based on these findings, we examined the biological activities of LW in human OA chondrocyte cultures stimulated with *IL-1*β. Cell viability, apoptosis, mitochondrial superoxide anion production, and the mRNA levels of antioxidant enzymes were analyzed. In addition, we measured the gene expression of type II collagen *(Col2a1), aggrecan*, the major cytokines, *MMPs*, and of a panel of *miRNA* involved in OA pathogenesis. Finally, the regulation of the nuclear factor (*NF*)-κ*B* pathway was examined.

## 2 Materials and methods

### 2.1 Sample collection and isolation of the cultures

Human OA cartilage samples were collected from femoral heads of five patients with hip OA according to American College of Rheumatology criteria (33), subjected to total arthroplasty, from the Orthopedic Surgery, Azienda Ospedaliera Universitaria Senese (Siena, Italy) (Supplementary Table S1). OA grades defined by Mankin degree, ranged from 3 to 7 (34).

After surgery, the fragments of cartilage were processed to isolate chondrocytes, as previously described (35). For growth and expansion, cells were cultured in Dulbecco's Modified Eagle Medium (DMEM) (Euroclone, Milan, Italy) with phenol red, contained 10% fetal bovine serum (FBS) (Euroclone, Milan, Italy), 2% penicillin/streptomycin (P/S) (Sigma-Aldrich, Milan, Italy). OA primary chondrocytes at the first passage after isolation (p0 and p1) were employed for the experiments. For each single experiment a cell culture from a unique donor was used.

### 2.2 Treatment procedure

Human OA chondrocytes were plated in 6-well dishes at a starting density of 1 × 10^5^ cells/well, until 85% confluence, in DMEM, 10% FBS, and 2% P/S. LW was provided by Levico Terme Health Resort (Levico Terme, Italy), directly collected from the natural spring, at the temperature of 11 °C, sealed and kept at 4 °C in the dark, and immediately sent to our laboratory. As soon as the samples arrived at our laboratory, they were analyzed for their organoleptic features. To guarantee the sterility of mineral water, the samples were accurately filtered using a 0.2 μm filter. Chemical and physical composition of sulfate-arsenical-ferruginous LW is reported in Supplementary Table S2.

LW was firstly diluted 1:10 in deionized water (DW) to achieve the concentration used in clinical practice for balneotherapy; the cells were incubated with DMEM powder medium partially substituted with LW, for 50% or 25%, in agreement with our previous study (17). The cells incubated with DMEM powder medium, dissolved in DW, served as control culture (CTRL). The treatment was applied for a period of 24 h and 48 h, based on the best obtained results in terms of viability. The pH of the culture medium was maintained between 7.0 and 7.4.

The treatment was performed in the presence or in absence of *IL-1*β, at the concentration of 10 ng/ml (Sigma-Aldrich, Milan, Italy), according to the concentrations employed in previous reports (11), and to our best data on viability and apoptosis; it was added after 2 h of preincubation with 50% or 25% of LW, and maintained for 24 h and 48 h. Supplementary Figure S1 showed results obtained in the presence of LW at a concentration of 50% for 24 h.

Moreover, for *NF-*κ*B* inhibition experiment, some cells were pre-incubated for 2 h with 1 μM *BAY 117082* (*NF-*κ*B* inhibitor, IKKα/β) (Sigma–Aldrich, Milan, Italy) and then treated with LW at 50% for 24 h.

### 2.3 Cell viability

The viability of the cells was evaluated by MTT assay (3-[4,4-dimethylthiazol-2-yl]-2,5-diphenyl-tetrazoliumbromide) (Sigma-Aldrich, Milan, Italy), as previously reported (35). The percentage of survival cells was evaluated as absorbance of studied sample/absorbance of control × 100. Results were referred to as OD units per 10^4^ cells.

### 2.4 Flow cytometry analysis

Apoptosis was measured through a commercial kit provided with annexin-V and propidium iodide (PI) probes (ThermoFisher Scientific, Milan, Italy). After treatment the cells were harvested, collected into cytometry tubes, and centrifuged. The pellet was resuspended in a working solution of annexin-V and PI, according to the manufacturer's instructions, and incubated at room temperature for 15 min in the dark.

The evaluation of mitochondrial superoxide anion production was carried out by using a commercial kit of MitoSOX Red probe (ThermoFisher Scientific, Milan, Italy). After treatment, the chondrocytes were incubated for 15 min at 37 °C in the dark with a solution of MitoSOX Red, according to the instructions. Cells were then harvested, collected into cytometry tubes, and centrifuged. Then the pellet was dissolved in saline solution before flow cytometry.

A total of 10,000 cells per assay were measured by the instrument both for apoptosis and mitochondrial superoxide anion assessment. For the detection of apoptotic cells, the analysis was conducted considering cells simultaneously stained and positive to each dye, and the results were reported as percentage of total apoptosis; for superoxide anion production the results were expressed as median fluorescence.

### 2.5 Quantitative Real-Time PCR analysis

Following treatment procedure, cells were collected, and total RNA was extracted using Trizol Reagent (Euroclone, Milan, Italy), according to the manufacturer's instructions.

Five hundred ng of RNA were reverse transcribed for target genes and *miRNA* by using specific commercial kits (Qiagen, Hilden, Germany). The obtained cDNA was processed through real-time PCR using specific commercial kits for SYBR Green assay (Qiagen, Hilden, Germany). The primers used for PCR reactions are reported in Supplementary Table S3.

All qPCR reactions were achieved in glass capillaries and processed by a LightCycler 1.0 (Roche Molecular Biochemicals, Mannheim, Germany) with LightCycler Software Version 3.5. For the analysis of the dissociation curves, an agarose gel was set up to observe the amplicon lengths and confirm the correct amplification of the resulting PCR products.

The Ct values of each sample were calculated and converted into relative expression for the data quantifications (36, 37). The normalization of the data was carried out using the housekeeping genes, *ACTB* for target genes and *SNORD-25* for *miRNA* (38).

### 2.6 Immunofluorescence analysis

Human OA chondrocytes were grown in coverslips mounted in multi-wells at a starting low density of 4 × 10^4^ cells/chamber, to avoid any possible overlapping. The cells were incubated for 4 h with 50% and 25% of LW, with or without *IL-1*β, the appropriate timing to evaluate the *NF-*κ*B* proteins. Afterwards, chondrocytes were fixed in 4% paraformaldehyde (ThermoFisher Scientific, Milan, Italy), permeabilized with a blocking solution, and incubated overnight at 4 °C with mouse monoclonal antip50 subunit primary antibody (Santa Cruz Biotechnology, Milan, Italy). These steps were followed by 1 h incubation with goat anti-mouse IgG-Texas Red conjugated antibody (Southern Biotechnology, Birmingham, AL, USA). Then, the coverslips were washed, and a nuclear colorant was added before mounting on specific slides.

Fluorescence was examined with a Leitz Aristoplan fluorescence microscope and the epifluorescence was analyzed at 200 × and 400 × magnification. About 100 cells for each experimental condition were randomly assessed and scored by the same operator. The fluorescent signal was evaluated as a fair, medium, or strong label, according to our previous study (39).

### 2.7 Statistical analysis

All assays were carried-out in three independent experiments for each of the samples collected and the results were expressed as the mean ± standard deviation (SD) of triplicate values for each experimental condition. The normal distribution of data was evaluated by Shapiro–Wilk, D'Agostino and Pearson, and Kolmogorov–Smirnov tests. MTT, flow cytometry, and immunofluorescence results were analyzed by ANOVA with Bonferroni *post-hoc* test; quantitative real time PCR was evaluated by oneway ANOVA with a Tukey's *post-hoc* test using 2^ΔΔ*CT*^ values for each sample. All tests were provided by the SAS System (SAS Institute Inc., Cary, NC, USA) and GraphPad Prism 6.1. (35). A *P*-value of < 0.05 was considered significant.

## 3 Results

### 3.1 Viability, apoptosis and redox balance

The effect of the treatment with 50% or 25% of LW, at different time points (0 h, 12 h, 24 h, 36 h, and 48 h), on the percentage of survival of OA chondrocytes are summarized in Supplementary Figure S2. The percentage of viability was not significantly modified by LW, at all-time points and concentrations tested. Thus, 24 h and 48 h were chosen as testing time points for the following experiments, in agreement with our previous study (35)

**Figure 1:**
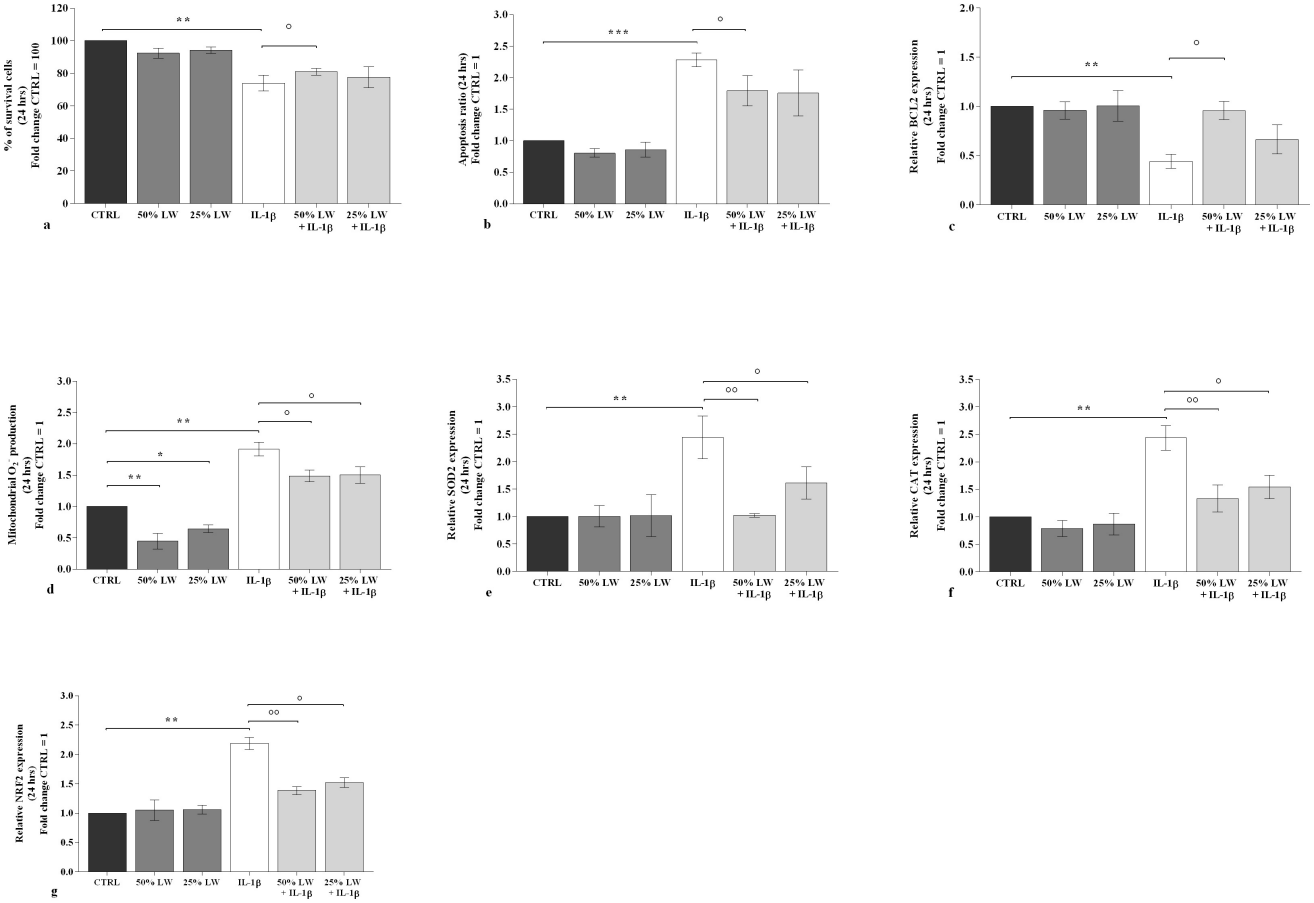
Chondrocytes were incubated for 24 h with Levico water (LW), at 50% or 25%, in the presence or not of interleukin (IL)-1β (10 ng/ml). **(a)** Evaluation of cell viability by MTT assay; **(b)** Detection of apoptosis by flow cytometry analysis; **(c)** Gene expression of B-cell lymphoma (BCL2) by quantitative real time PCR; **(d)** Mitochondrial superoxide anion production by flow cytometry; **(e-g)** Gene expression of antioxidant enzymes by quantitative real time PCR. The data analysis was calculated as fold change to control culture (CTRL = equal to 100 or 1). Data were represented as mean ± standard deviation. *P < 0.05, **P < 0.01, ***P < 0.001 versus CTRL; *P* < 0.05, *P* < 0.01 versus *IL-1*β.

**Figure 2:**
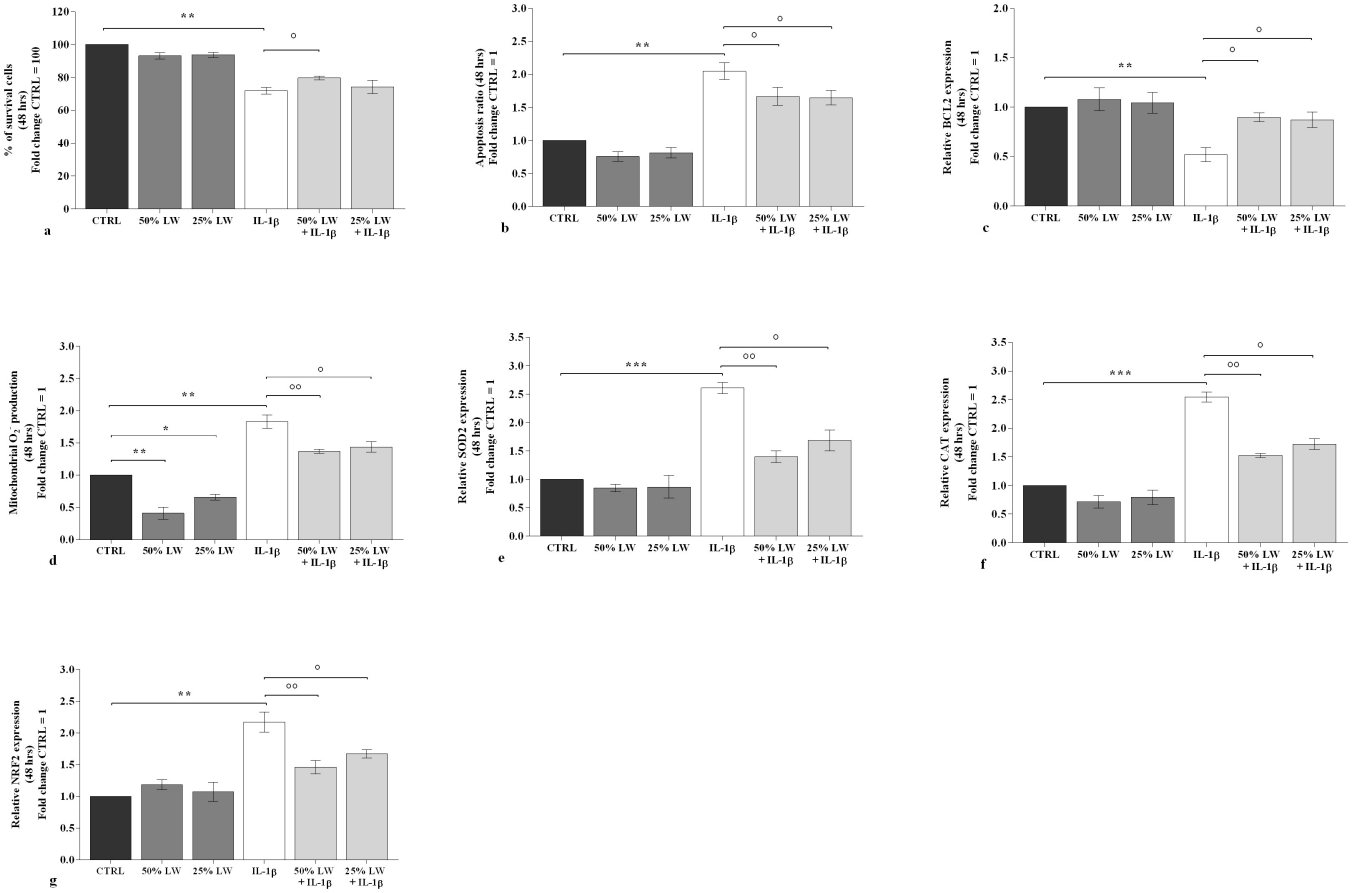
Chondrocytes were incubated for 48 h with Levico water (LW), at 50% or 25%, in the presence or not of interleukin (IL)-1β (10 ng/ml). **(a)** Evaluation of cell viability by MTT assay; **(b)** Detection of apoptosis by flow cytometry analysis; **(c)** Gene expression of B-cell lymphoma (BCL2) by quantitative real time PCR; **(d)** Mitochondrial superoxide anion production by flow cytometry; **(e-g)** Gene expression of antioxidant enzymes by quantitative real time PCR. The data analysis was calculated as fold change to control culture (CTRL = equal to 100 or 1). Data were represented as mean ± standard deviation. *P < 0.05, **P < 0.01, ***P < 0.001 versus CTRL; *P* < 0.05, *P* < 0.01 versus *IL-1*β.

The incubation of the cells with 50% or 25% of LW alone, for 24 h and 48 h, did not induce any significant change on viability and apoptosis in comparison to CTRL (Figures 1a, b, 2a, b).

*IL-1*β significantly reduced viability (*P* < 0.01), induced apoptosis (*P* < 0.001 at 24 h and *P* < 0.01 at 48 h) and decreased the gene expression of the anti-apoptotic marker, B-cell lymphoma (*BCL2*) (*P* < 0.01), which were all counteracted by the pre-treatment of the cells with LW at 50% (*P* < 0.05) (Figures 1a–c, 2a–c). Furthermore, 25% of LW resulted in being able to significantly reverse the *IL-1*β effects on the apoptosis ratio and *BCL2* mRNA at 48 h of treatment (*P* < 0.05) (Figure 2b, c).

To investigate the potential role of LW in regulating chondrocytes oxidant/antioxidant balance, we performed an analysis of mitochondrial superoxide anion production and of the gene expression of superoxide dismutase (*SOD)2*, catalase (*CAT*) and nuclear factor erythroid 2 like 2 (*NRF2*). Our results showed that 50% or 25% of LW, tested alone at 24 h and 48 h, significantly reduced the production of mitochondrial superoxide anion in comparison to CTRL, in a dose-dependent manner (*P* < 0.05, *P* < 0.01); while no significant modifications of gene expression of the analyzed antioxidant enzymes were found (Figures 1d–g, 2d–g). Conversely, *IL-1*β induced a significant increase of mitochondrial superoxide anion production (*P* < 0.01) and of the expression of the antioxidant enzymes (*P* < 0.01 at 24 h; *P* < 0.001 at 48 h, for *SOD2* and *CAT*; *P* < 0.01 for *NRF2*) (Figures 1e–g, 2e–g). This negative trend resulted significantly attenuated by the presence of 50% or 25% of LW and dose-dependent, except for superoxide anion production at 24 h (Figures 1d–g, 2d-g).

### 3.2 Gene expression of cytokines, metalloproteinases, Col2a1 and aggrecan

**Figure 3:**
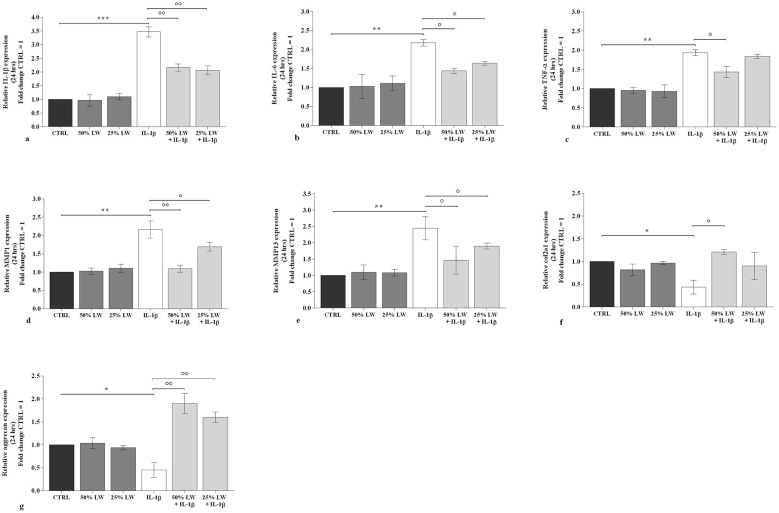
Chondrocytes were incubated for 24 h with Levico water (LW), at 50% or 25%, in the presence or not of interleukin (IL)-1β (10 ng/ml). **(a-g)** Gene expression of IL-1β, IL-6, tumor necrosis factor (TNF)-α, metalloproteinase (MMP) 1 and MMP 13, type II collagen (Col2a1), and aggrecan by quantitative real time PCR. The data analysis was calculated as fold change to control culture (CTRL = equal to 1). Data were represented as mean ± standard deviation. *P < 0.05, **P < 0.01, ***P < 0.001 versus CTRL; *P* < 0.05, *P* < 0.01 versus IL-1β.

**Figure 4:**
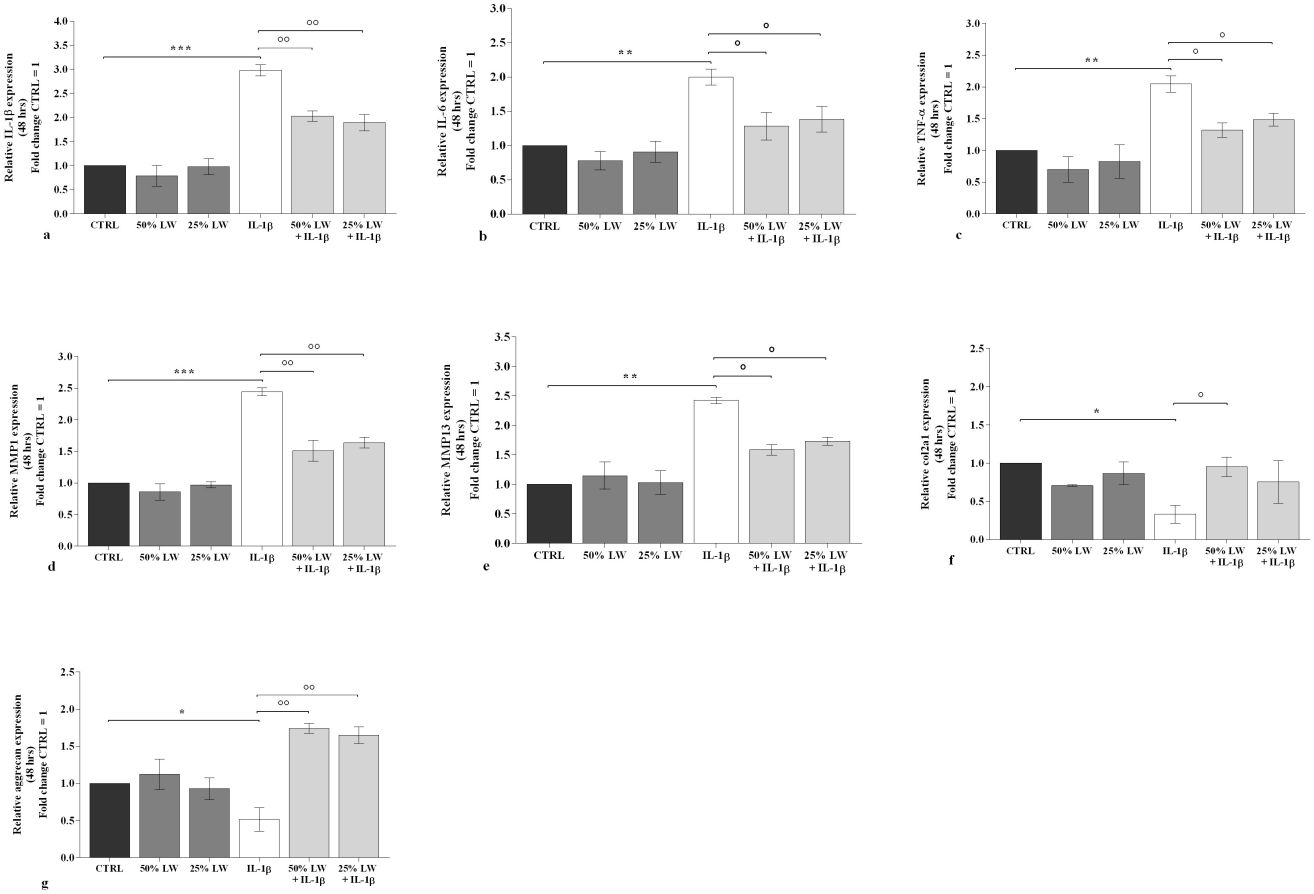
Chondrocytes were incubated for 48 h with Levico water (LW), at 50% or 25%, in the presence or not of interleukin (IL)-1β (10 ng/ml). **(a-g)** Gene expression of IL-1β, IL-6, tumor necrosis factor (TNF)-α, metalloproteinase (MMP) 1 and MMP 13, type II collagen (Col2a1), and aggrecan by quantitative real time PCR. The data analysis was calculated as fold change to control culture (CTRL = equal to 1). Data were represented as mean ± standard deviation. *P < 0.05, **P < 0.01, ***P < 0.001 versus CTRL; *P* < 0.05, *P* < 0.01 versus IL-1β.

As shown in Figure 3 and in Figure 4, 50% or 25% of LW, tested alone, didn't modify the transcriptional levels of *IL-1*β, *IL-6*, and tumor necrosis factor (*TNF)-*α, at both time-points, as compared to controls. A similar trend was observed for the expression of *MMP-1* and *MMP-13, Col2a1*, and *aggrecan*. On the other hand, a significant increase of the pro-inflammatory cytokines and *MMP*s and a down-regulation of *Col2a1* and *aggrecan* gene expression were shown in chondrocyte cultures stimulated with *IL-1*β (Figure 3 and Figure 4). The pre-incubation of the cells with 50% of LW inhibited the proinflammatory and prodegrading effects of *IL-1*β, for all the analyzed target genes at 24 and 48 h (*P* < 0.05, *P* < 0.01). 25% of LW was able to significantly counteract *IL-1*β activities on these markers (*P* < 0.05, *P* < 0.01), except for *Col2a1 and TNF-*α in the observation at 24 h (Figure 3c, f) and *Col2a1* at 48 h (Figure 4f).

### 3.3 Gene expression of miRNA

**Figure 5:**
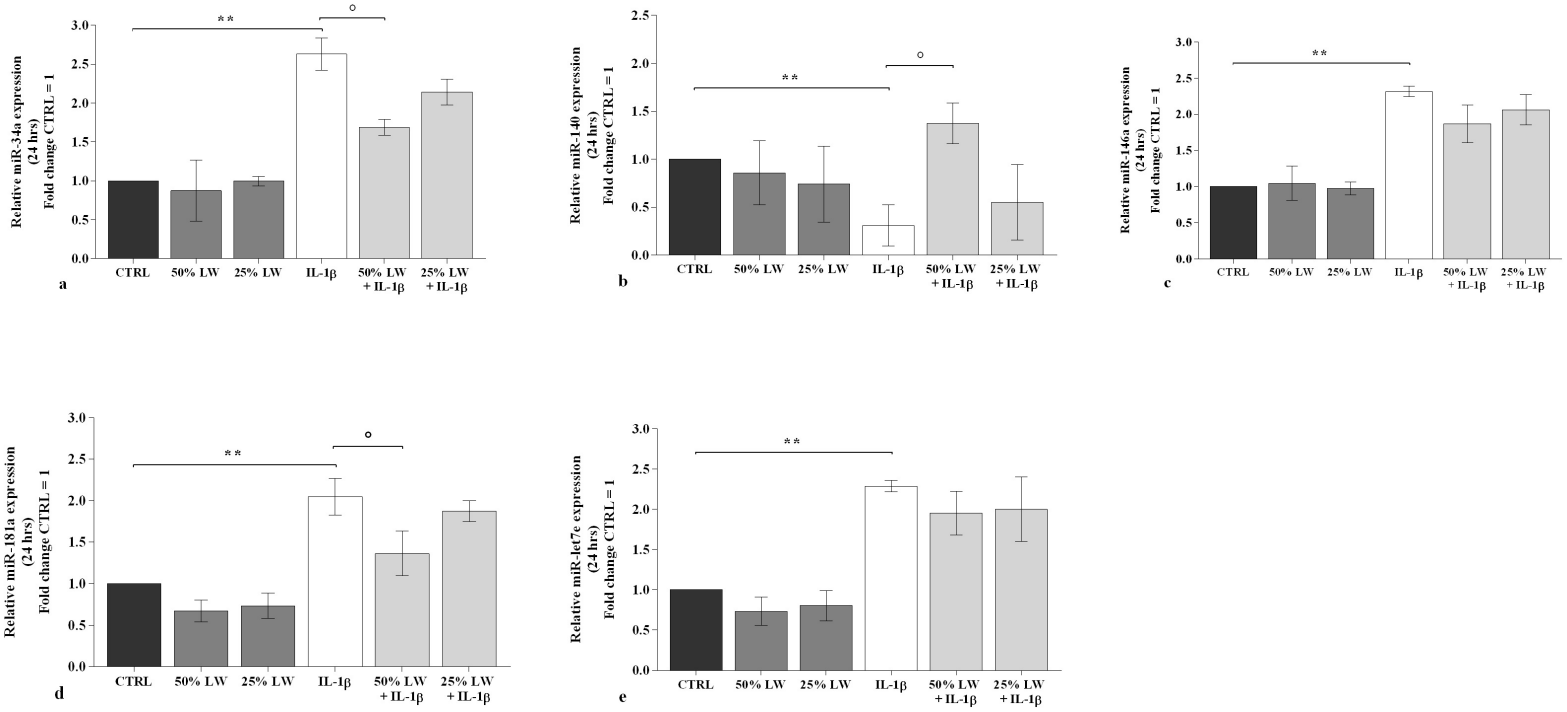
Chondrocytes were incubated for 24 h with Levico water (LW), at 50% or 25%, in the presence or not of interleukin (IL)-1β (10 ng/ml). **(a-e)** Gene expression of microRNA (miRNA) by quantitative real time PCR. The data analysis was calculated as fold change to control culture (CTRL = equal to 1). Data were represented as mean ± standard deviation. **P < 0.01 versus CTRL; *P* < 0.05 versus IL-1β.

**Figure 6:**
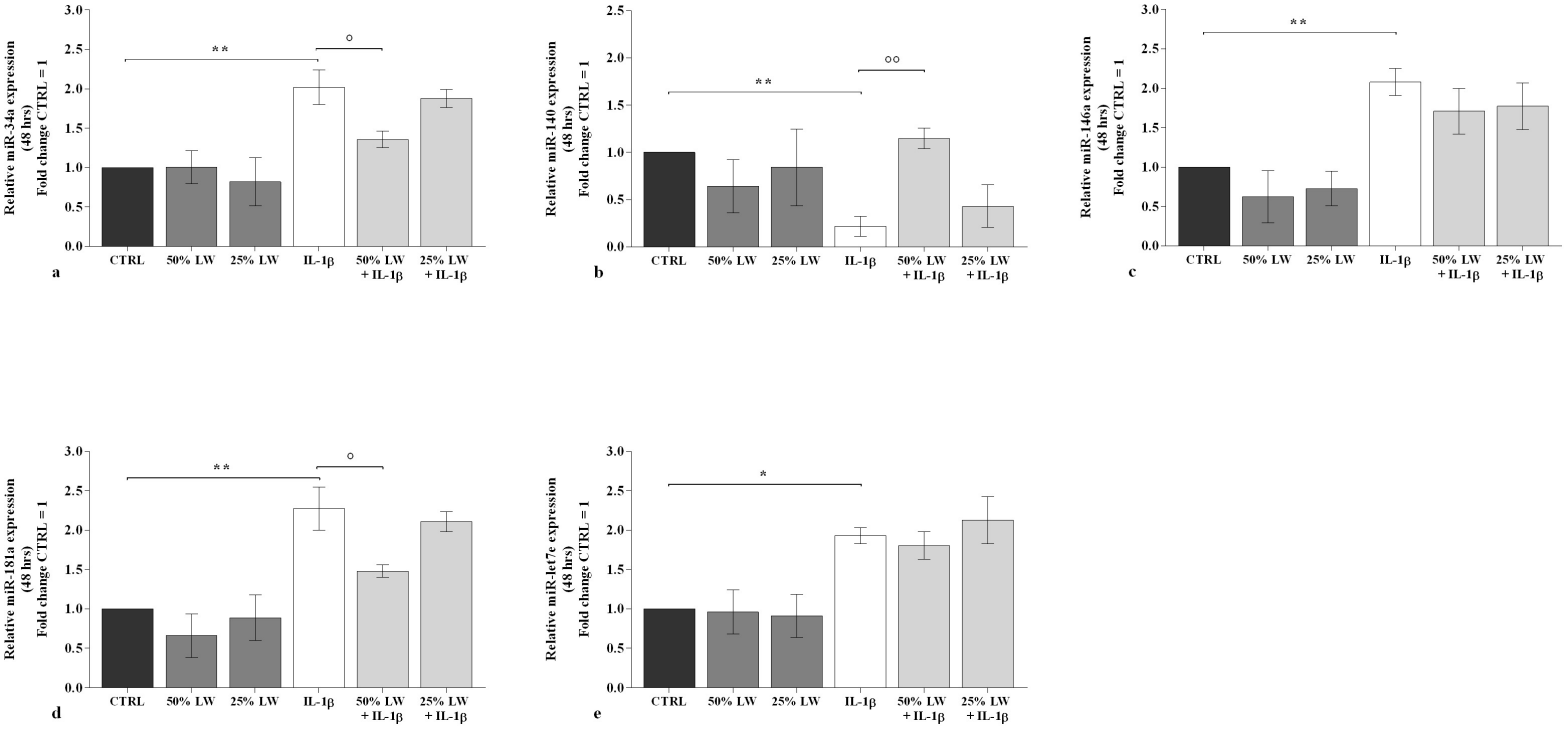
Chondrocytes were incubated for 48 h with Levico water (LW), at 50% or 25%, in the presence or not of interleukin (IL)-1β (10 ng/ml). **(a-e)** Gene expression of microRNA (miRNA) by quantitative real time PCR. The data analysis was calculated as fold change to control culture (CTRL = equal to 1). Data were represented as mean ± standard deviation. *P < 0.05, **P < 0.01 versus CTRL; *P* < 0.05, *P* < 0.01 versus IL-1β.

LW at 50% or 25% tested alone, did not alter the transcriptional levels of the considered *miRNA* (Figure 5, Figure 6). *IL-1*β significantly up-regulated the gene expression of *miR-34a, miR-146a, miR-181a*, and *miR-let-7e*, while it reduced that of *miR-140* (*P* < 0.01), at 24 h and 48 h time points. These effects were significantly reduced by the pre-incubation of chondrocytes with 50% of LW, for the expression of *miR-34a, miR-140*, and *miR-181a*, at the studied time points. Conversely, LW at 25% seemed not to be equally relevant in limiting *IL-1*β effects on *miRNA* profile (Figure 5, Figure 6).

### 3.4 Immunofluorescence analysis and gene expression of NF-κB subunits

To elucidate the potential mechanism by which LW might regulate the observed effects, we evaluated the possible involvement of *NF-*κ*B* pathway.

**Figure 7:**
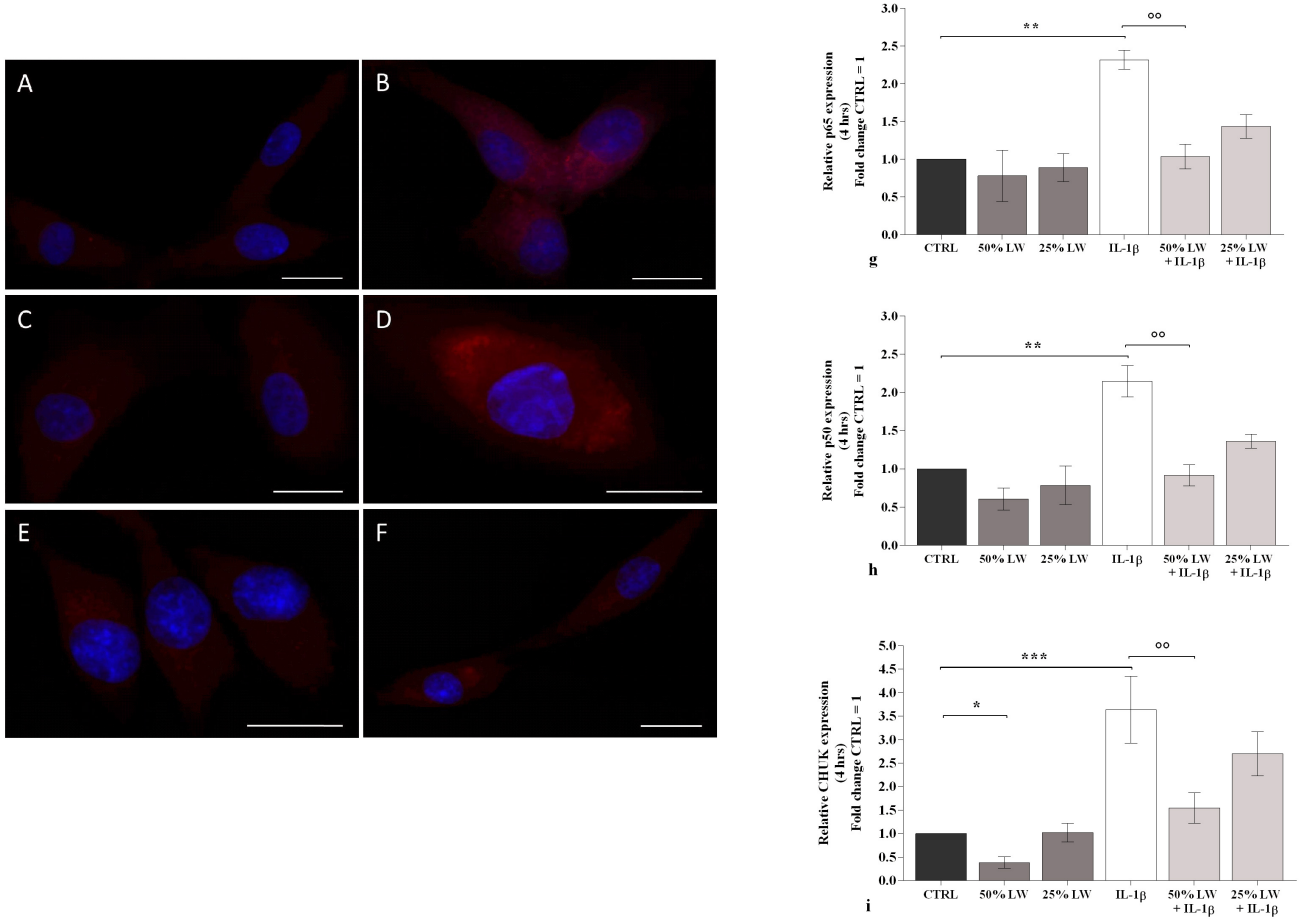
Chondrocytes were incubated for 4 h with Levico water (LW), at 50% or 25%, in the presence or not of interleukin (IL)-1β (10 ng/ml). **(A-F)** Indirect immunofluorescence microscopy of monoclonal anti-p50 subunit primary antibody. Control culture (CTRL): a fair fluorescence in the cytoplasm is shown **(A)**; IL-1β: an intense signal is evident in the cytoplasm **(B)**; 25% LW **(C)** and 25 LW+ IL-1β **(D)**: the label is almost absent in **(C)** and strongly present in **(D)**; 50% LW **(E)** and 50 LW+ IL-1β **(F)**: the signal is like CTRL in **(E)** but appears diffused in **(F)**. Nuclei (blue). Bars: A, F 20 μm; B, C 30 μm; D, E 40 μm. **(g-i)** Gene expression of nuclear factor (NF)-κB subunits by quantitative real time PCR. The data analysis was calculated as fold change culture (CTRL = equal to 1). Data were represented as mean ± standard deviation. *P < 0.05, **P < 0.01, ***P < 0.001 versus CTRL; *P* < 0.01 versus IL-1β.

An immunofluorescence assay was performed to investigate the cytoplasmic and nuclear signal intensity of the p50 NF-κB subunit in cultures treated with 25% and 50% LW, with or without IL-1β, for 4 h. As described in the Materials and Methods section, p50 *NF-*κ*B* labeling was categorized as weak, medium, or strong (Table 1) across the different experimental conditions. Figure 7 illustrates the most representative labeling for each treatment.

**Table 1:** Immunofluorescence for p50 nuclear factor (NF)-κB was analyzed and classified into three categories based on signal intensity: weak, medium, or strong. Results are presented as the percentage of cells for each staining category across the different experimental conditions.

p50 *NF-κB intensity*	Weak signal %	Medium signal %	Strong signal %
CTR	90	5	10
CTR + *IL-1β*	5	10	85
25% of LW	85	10	5
25% of LW+ *IL-1β*	5	10	85
50% of LW	85	10	5
0% of LW+ *IL-1β*	10	55	35

In control cultures, the p50 *NF-*κ*B* signal was low and primarily localized within the cytoplasm, with minimal nuclear translocation (Figure 7A). Stimulating the cells with *IL-1*β led to a significant increase in the p50 signal, characterized by higher cytoplasmic intensity and enhanced nuclear translocation compared to CTRL (Table 1, Figure 7B). No relevant changes in signal intensity were observed following treatment with 25% LW alone (Figure 7C) or in combination with *IL-1*β (Figure 7D) when compared to the respective controls.

In contrast, the 50% LW concentration significantly changed this trend (Table 1, Figure 7E). Specifically, pre-treatment with 50% LW effectively counteracted the effects of *IL-1*β, leading to a significant reduction in the percentage of cells exhibiting a strong signal (Table 1, Figure 7F).

The real-time PCR analysis of p50, p65, and of the inhibitor of nuclear factor kappa-B kinase subunit alpha (*IKK-*α*, Chuk*) showed that the incubation of OA chondrocytes with 50% or 25% of LW, tested alone, did not modify the mRNA levels of p65 and p50 subunits, while 50% of LW significantly reduced the expression of *Chuk* (*P* < 0.05) (Figure 7g-i). As expected, a significant increase of target genes expression was induced by *IL-1*β (*P* < 0.01) when compared to the CTRL cultures, while the pre-incubation of the cells with 50% of LW significantly limited this trend (*P* < 0.01); 25% of LW reduced the effects of *IL-1*β, even if not in a significant manner (Figure 7g-i).

### 3.5 Gene Expression of cartilage markers and miRNA after NF-κB Inhibition

Based on the previous reported results, and to confirm the possible role of *NF-*κ*B* pathway in mediating LW induced activity, OA chondrocytes were pre-treated with a specific *NF-*κ*B* inhibitor (*BAY 11–7082, IKK*α*/*β), and then, incubated with 50% of LW for 24 h.

**Figure 8:**
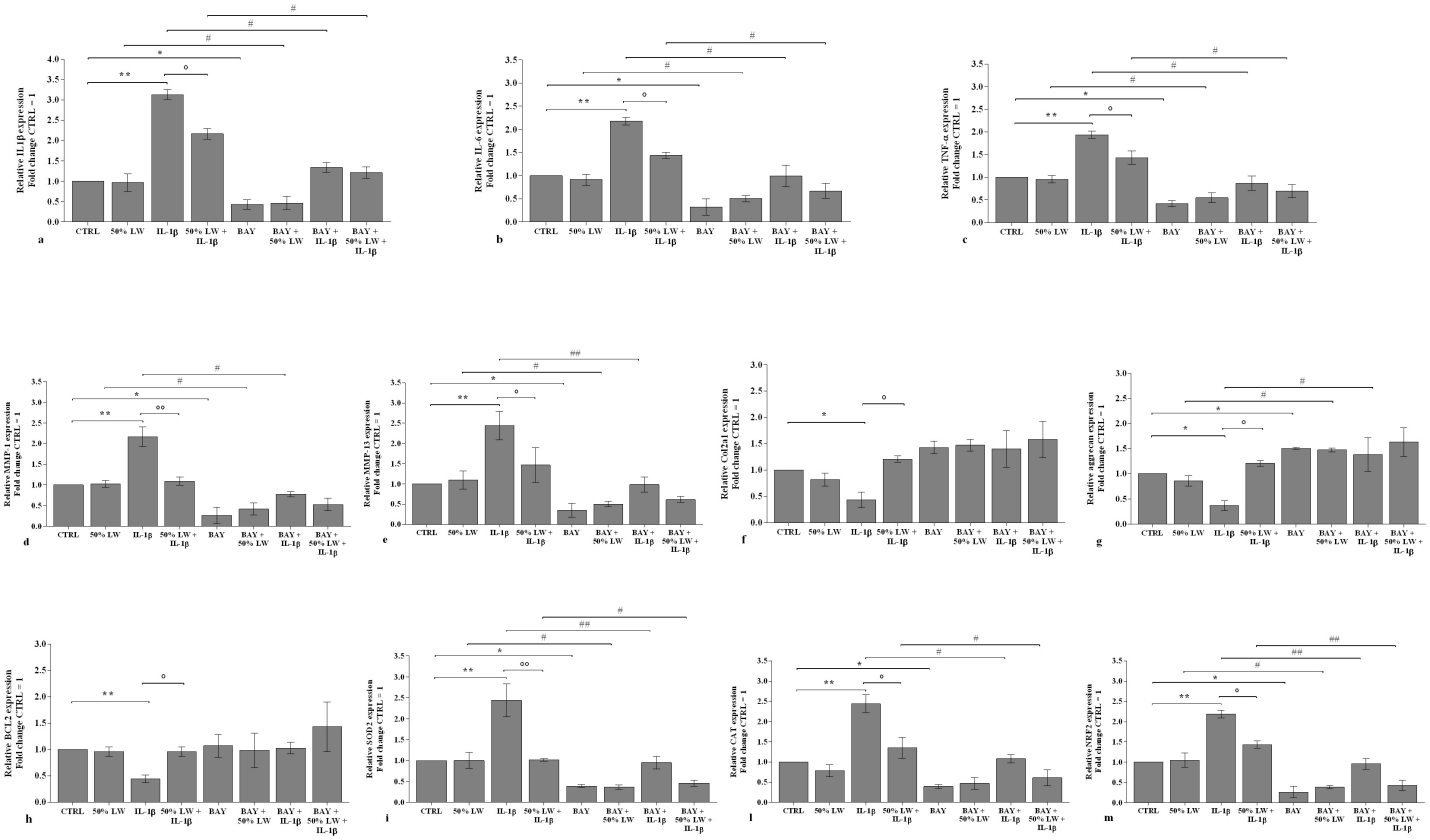
Chondrocytes were incubated for 24 h with Levico water (LW), at 50%, in the presence or not of interleukin (IL)-1β (10 ng/ml) and of NF-κB inhibitor (BAY 11–7082) (1 μM), (2 h of pretreatment). **(a-m)** Gene expression of cartilage markers by quantitative real time PCR. The data analysis was calculated as fold change to control culture (CTRL = equal to 1). Data were represented as mean ± standard deviation. *P < 0.05, **P < 0.01 versus CTRL; *P* < 0.05, *P* < 0.01 versus IL-1β; # *P* < 0.05, ## *P* < 0.01 versus BAY.

**Figure 9:**
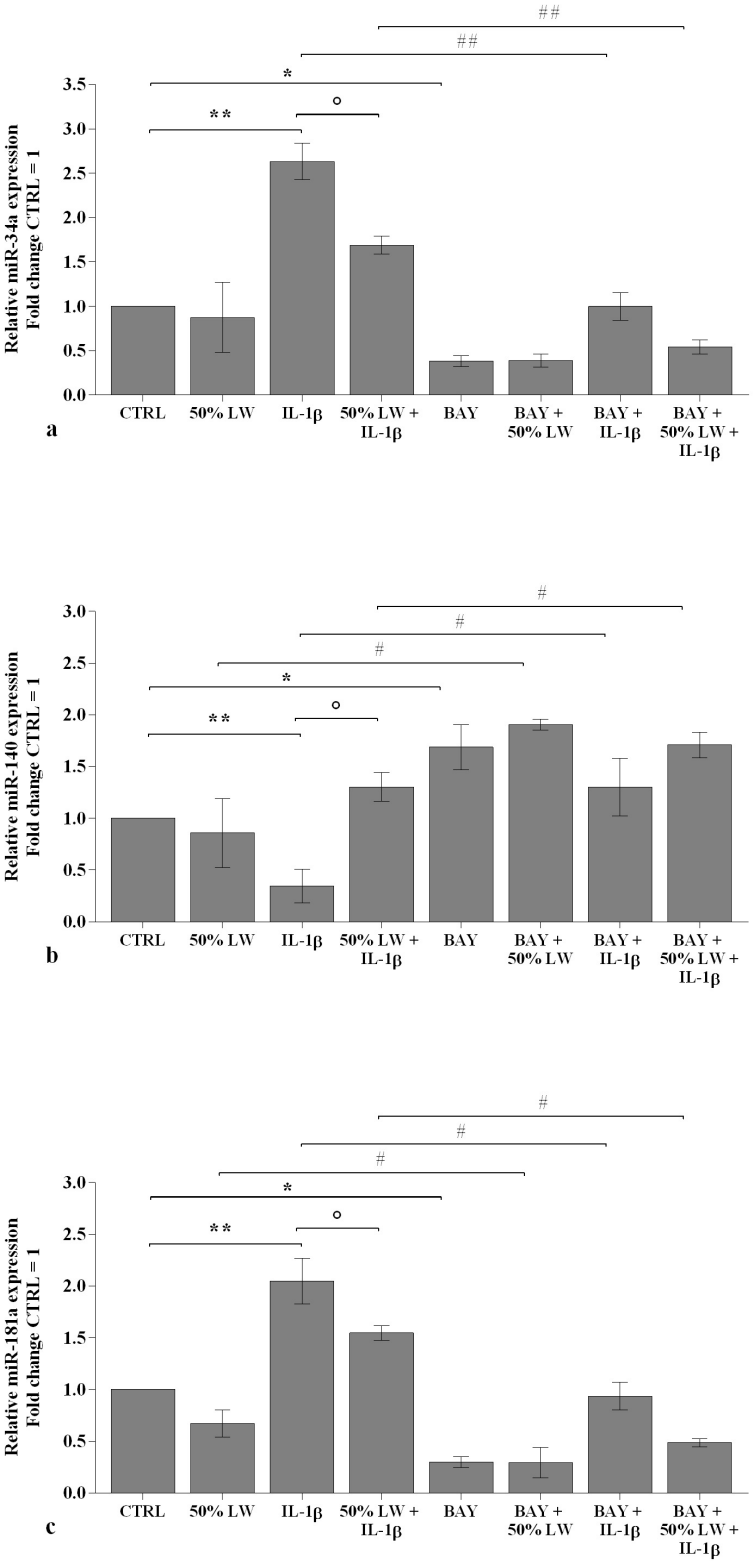
Chondrocytes were incubated for 24 h with Levico water (LW), at 50%, in the presence or not of interleukin (IL)-1β (10 ng/ml) and with 1 μM of NF-κB inhibitor (BAY 11–7082) (2 h of pretreatment). **(a-c)** Gene expression of microRNA (miRNA) by quantitative real time PCR. The data analysis was calculated as fold change to control culture (CTRL = equal to 1). Data were represented as mean ± standard deviation. * *P* < 0.05, **P < 0.01 versus CTRL; *P* < 0.05 versus IL-1β; # *P* < 0.05, ## *P* < 0.01 versus BAY.

The transcriptional levels of *IL-1*β*, IL-6, TNF-*α*, MMP-1, MMP-13, SOD-2, CAT, NRF2* (Figure 8), *miR-34a*, and *miR-181a* (Figure 9) were significantly down-regulated (*P* < 0.05) in OA cells incubated with *BAY 11–7082*, in comparison to CTRL, while an increase of *aggrecan* (*P* < 0.05, Figure 8g) and *miR-140* levels was observed (*P* < 0.05, Figure 9b). The presence of *NF-*κ*B* inhibitor significantly limited the effect caused by *IL-1*β on all the analyzed genes (*P* < 0.05, *P* < 0.01) (Figure 8, Figure 9).

The co-treatment of the cells with *BAY 11–7082* and 50% of LW did not show any difference in the expression of the studied targets with respect to the only incubation with *BAY 11–7082* (Figure 8, Figure 9). Surprisingly, this concomitant incubation significantly enhanced the regulation on the studied genes in comparison to the effects induced by LW alone, while it reduced the negative effects caused by *IL-1*β (Figure 8, Figure 9).

No modulation of *Col2a1* and *BCL2* expression levels was triggered by *BAY 11–7082* (Figure 8f, h).

## 4 Discussion

Several studies have corroborated the therapeutic effects of the sulfate-arsenical-ferruginous LW in musculoskeletal conditions, which are partially ascribable to its thermal and mechanical effects, and in part, to its chemical composition (2–6).

In this preliminary study, we examined the potential biological properties of LW, tested as a whole, in human OA chondrocyte cultures. According to our previous findings (17) the cells were cultivated with LW, at 50% and 25% of concentrations. The treatment was applied for 24 h and 48 h based on the best obtained results in terms of viability of chondrocytes and was performed at 37 °C. The exposure of the cells to different temperatures significantly affects their survival and activity ((40–42)).

Under these experimental conditions, we evaluated apoptosis, mitochondrial superoxide anion production and the gene expression of the antioxidant enzymes and anti-apoptotic marker *BCL2*. Furthermore, we analyzed the expression of markers of cartilage turnover, and of major cytokines, *MMPs*, and *miRNA* that significantly affect the pathophysiology of OA.

Apoptosis and oxidative stress are important hallmarks of OA. The failure of the oxidant/antioxidant balance in chondrocytes, primarily due to defects in mitochondrial function, induces a persistent alteration in redox status, which promotes cartilage degradation and inflammation (43, 44). Increased *ROS* generation also makes cells more susceptible to death through apoptosis by dysregulation of the release of pro- and anti-apoptotic proteins in the cytosol (44).

The analysis of viability and apoptosis carried-out in our study confirmed a reduction in survival percentage and an increase in apoptotic chondrocytes following treatment with *IL-1*β, in agreement with the existing literature (45). *IL-1*β also activates oxidative stress, which increases the production of mitochondrial superoxide anion and the expression of the antioxidant enzymes, *SOD2* and *CAT*, and the transcriptional factor *NRF2*, which is consistent with our previous experience (35). Interestingly, we observed that 50% and 25% LW restricted the effect of *IL-1*β activity on viability, apoptosis, and redox balance, and promoted survival, by limiting apoptosis and reducing the activation of oxidative stress. The results are consistent with our study performed with Vetriolo water (25% and 50% concentration) in human OA chondrocytes, in which we observed enhanced viability, reduced apoptosis, and decreased *NO* release and *iNOS* protein expression induced by *IL-1*β stimulation (17). This effect was partially associated with the presence of iron in the water. The ability of ferric iron (Fe^3+^) to reduce *NO* release, *iNOS* activity, and expression was demonstrated previously in macrophage-like cells (46). In turn, *NO* regulates apoptosis, through an imbalance in the release of pro- and anti-apoptotic proteins (43, 45, 47). Recently, other investigators using human lung fibroblasts to assess mineral waters from three Spanish Health Resorts with a high content of chloride, sodium, and sulfate, reported a significant increase in cell proliferation and an enhanced antioxidand capacity (19). Lately, Vaz et al. (14) analyzed the potential anti-senescence and antioxidant properties of several Portuguese thermal waters in murine skin fibroblast cell lines observing anti-aging activity of sulfated/calcic mineral waters, which decreased senescence and preserved viability. These findings prompted us to hypothesize that the presence of iron and/or sulfate, may be responsible for the observed results in terms of viability, apoptosis, and oxidative stress (46, 48, 49).

It is well established that the increased release and expression of proinflammatory cytokines and *MMPs*, promote the progressive destruction of articular cartilage and represent the major hallmark of the OA process (23).

In the present study, we confirmed an up-regulation of gene expression of some pro-inflammatory cytokines, as *IL-1*β*, IL-6*, and *TNF-*α, in OA chondrocytes stimulated with *IL-1*β, which was consistent with other reports (50, 51). We also observed the ability of LW to counteract the *IL-1*β-induced effects on cytokine expression. Other groups have demonstrated the beneficial effects of various mineral waters on proinflammatory cytokines, although there were differences in the concentration and chemical composition of the waters analyzed and in the cell cultures employed. Three studies on human psoriatic keratinocytes evaluated Comano mineral water (Comano Terme, Italy), containing sodium, calcium, and bicarbonate, and demonstrated its ability to reduce the expression and release of *IL-8, IL-6*, and *TNF*α (16, 52, 53). In a study on human keratinocyte cell lines, Lee et al. (54) showed that the spa spring water of Yong-gung oncheon (Gangwha-gun, Korea), which is rich in sulfur, magnesium, and calcium, reduced the expression of *IL-1*α*, IL-6*, and *TNF-*α. More recently, the anti-inflammatory effect of a sulfated-ferruginous mineral water from the thermal springs of Châtel-Guyon (Chatel-Guyon, France) was observed in a human intestinal epithelial cell line through the reduction of *IL-8* (55). Similarly, hydrogen sulfide reduces expression and secretion of *IL-8* in human keratinocytes cultures and interferes with *IL-17* and *IL-22*-induced *IL*-8 production (56). Finally, in a keratinocyte model (HaCaT), Zoller et al (57) showed the ability of two thermal mineral waters (La Roche Posay and Avène*)* to inhibit the production of *IL-6*.

We also observed the upregulation of *MMP-1* and *MMP-13* expression, and a concomitant downregulation of *aggrecan* and *Col2a1* in OA chondrocytes stimulated with *IL-1*β, as previously reported (50, 51). Interestingly, we found that LW limited the negative effects of *IL-1*β; thus, we firstly demonstrated the potential of LW to regulate cartilage turnover altered by this cytokine.

During the last decade, *miRNA* have been studied for their role in OA pathogenesis (25–28). They represent a class of endogenous conserved noncoding RNA molecules of 22–25 nucleotides and are important posttranscriptional regulators of gene expression. *MiRNA* exert their biological effects by suppressing the translation of target genes, thus playing an important role in various cellular processes (58, 59). The results from *in vitro* studies suggest beneficial and detrimental effects of *miRNA* during OA damage, which indicates their activity in regulating cartilage metabolism, inflammation, apoptosis, and oxidative stress (35, 60, 61).

In the present study, we examined the potential effects of LW in regulating the gene expression of a pattern of *miRNA*, including *miR-34a, miR-140, miR-146a, miR-181a*, and *miR-let7e*, which play an important role in OA development **(**59, 61, 62). As expected, our results showed that *IL-1*β significantly modified the expression of these *miRNA*, increasing the levels of *miR-34a, miR-146a, miR-181a*, and *miR-let7e*, while downregulating *miR-140*, which is consistent with previous reports (62–66).

Intriguingly, we demonstrated the ability of 50% LW to counteract the dysregulation of *miR-34a, miR140*, and *miR-181a* expression induced by *IL-1*β, whereas 25% of LW was not equally effective at attenuation *miRNA* expression. Thus, we hypothesized that the lower degree of mineralization may not be sufficient to exert a significant biological response on *miRNA*. Taken together, the obtained results indicate the protective role of LW considering that *miR-34a*, and *miR-181a* are upregulated in OA and have a key role in its pathogenesis, by increasing apoptosis and generating *ROS* and *NO* (67–70). In contrast, the reduced expression of *miR-140* has been reported in OA chondrocytes compared with that in normal cells (71). Moreover, *miR-140* stimulates cartilage matrix formation, inhibits inflammation and cartilage degeneration, and therefore represents a potential therapeutic target for OA (72, 73). Finally, the capacity of LW in modulating the expression of some *miRNA* implicated in OA pathogenesis, corroborates our previous clinical data in patients with gonarthrosis who received a cycle of balneotherapy (29). Currently, it is challenging to explain the ability of LW to regulate the expression of *miRNA*, as observed in our *in vitro* model, due to the lack of similar literature reports. Recently, Banarjee et al. (74) demonstrated that the chronic exposure of HaCaT cells to increasing concentrations of arsenic, for up to 28 weeks, induced a dysregulation of the gene expression of some *miRNA* and their target genes, probably through the modulation of *NRF2* transcription factor (74).

Finally, we determined the potential involvement of the *NF-*κ*B* signaling pathway in the regulation of the biological effects of LW. The *NF-*κ*B* pathway is the most important signaling mechanism associated with the inflammatory and degrading processes in OA (75–77). *NF-*κ*B* is a ubiquitous protein that specifically binds to DNA consensus sequences to induce transcription. This factor exists in the cytoplasm in an inactive form bound to its inhibitor *I*κ*B*. Various extracellular stimuli, including proinflammatory mediators, such as *IL-1*β, activate *NF-*κ*B* through the interaction of the catalytic subunit *IKK-*α *(Chuk)* with *I*κ*B* (78, 79). IKK-α induces the phosphorylation of *I*κ*B* and its subsequent degradation, thus enabling the *NF-*κ*B* subunits to migrate into the nucleus and recognize specific promoter sequences of genes involved in inflammation, cartilage matrix degradation, and apoptosis (75–77).

In the present experience, we observed the activation of the *NF-kB* signal after stimulating OA chondrocytes with *IL-1*β by an increase of the gene expression of *Chuk, p50* and *p65* subunits, as well as through the activation and nuclear translocation of *p50* protein, according to our previous study (80). The preincubation of cells with LW neutralized the activation of *NF-kB* induced by *IL-1*β. These results were dose-dependent and strongly enhanced at higher mineral water concentrations.

In the current study, we used 1 μM of BAY11–7082, a specific inhibitor of *NF-*κ*B* activation, as reported in other studies on chondrocyte cultures (35, 50, 81). The co-treatment of the cells with BAY11–7082 reduced the effects of *IL-1*β, as previously reported (35). Interestingly, it potentiated the activity of LW on all analyzed parameters. These results suggest that LW may be effective in regulating chondrocyte activity through a possible inhibition of NF-κB signaling.

The clinical efficacy of balneotherapy in musculoskeletal disorders, such as OA, is the result of a combination of physical, chemical, and microbiological factors (7). The chemical properties of medicinal mineral waters or muds are important in achieving the beneficial effects beside thermal stress as supported, albeit indirectly, by studies comparing the effectiveness of natural mineral waters or mud-packs to treatments lacking chemical component, such as for example tap water or mud depleted of minerals or applied over an impermeable stretch film, ect) (3, 82–86). In addition, the “chemical hypothesis” is further corroborated by the persistence of beneficial clinical effects over time, as described in long-term studies (3, 87–89).

Moreover, it is plausible that the therapeutic activity of balneotherapic treatments is related to a complex relationship among a number of different mineral elements rather than to a single component (7, 82). On the basis of this consideration the present research focused on the biological effects of a mineral water as a whole; therefore the evaluation of the effects of single mineral compound that characterize LW was beyond the scope of this study. The results presented here may therefore be contradictory to the evaluations carried out with single mineral elements (90–92).

Finally, the problems related to the absorption of the mineral compounds through the skin and, thus, into the circulation, remain to be elucidated (93). At this regard, data from the literature are still scarce or derived mainly from pre-clinical models. Among them, *in vitro* studies have been performed on human cutaneous samples and have demonstrated the transdermal migration of different chemical elements (94–96). In addition, Shani et al. (97) found traces of mineral elements in the serum of Guinea-pigs and of psoriatic patients after daily bathing in the Dead-Sea.

In conclusion, the results of this preliminary study demonstrated, for the first time, the potential antiinflammatory activity of LW in human OA chondrocyte cultures through a reduction in apoptosis, mitochondrial superoxide anion production, and a regulation of gene expression of the main proinflammatory and degrading cartilage mediators. We also showed the potential role of the studied mineral water in modulating the transcriptional levels of *miR-34a, miR-140*, and *miR-181a*, which have been implicated in the pathogenesis of OA. Finally, it can be hypothesized that LW can exert its functions in chondrocytes via *NF-*κB pathway. Overall, our data provide new insight into the possible mechanisms underlying the efficacy of balneotherapy with a sulfate-arsenical-ferruginous mineral water in OA.

Nevertheless, our study had several limitations. First, gene expression using quantitative real-time PCR was the only technique performed in this report to assess the biological activities of LW. Western blot analysis of the protein levels of the candidate and target genes will be necessary to confirm that the changes occurring at the mRNA level affected protein modification, because they are not always correlated. Furthermore, to more deeply elucidate the effects of LW on redox balance it will be important to evaluate other indicators of oxidative stress at protein level or enzymatic activity (98). In addition, to confirm a role for *miRNA* in mediating the beneficial effects of LW, transfection experiments with *miRNA* inhibitors should be performed. Moreover, we cannot exclude the importance of microbial communities present in LW which may be responsible for its therapeutic effect against OA, in combination with mineral elements and mechanical and thermal stimuli. This aspect cannot be taken into account in this paper due to the filtration procedure applied for our experiments, responsible for removing water's microbiome.

Finally, it is important to highlight that we only conducted *in vitro* experiments; thus, they cannot be directly translated to an *in vivo* scenario, particularly because of limited knowledge regarding the absorption of mineral elements through the skin and the lack of data related to their concentration in the joint cavity.

## Data Availability

The data used to support the findings of this study are available from the corresponding author upon reasonable request. Requests to access these datasets should be directed to fioravanti7@virgilio.it.
