# Supplementary material for: A sulfate-arsenical-ferruginous water affects apoptosis, oxidative stress and the gene expression of inflammatory mediators and of a panel of MicroRNA in IL-1β stimulated human osteoarthritic chondrocytes
Source: Front Med (Lausanne). 2026 Apr 20;13:1800406. doi: 10.3389/fmed.2026.1800406 (PMC13137369; doi:10.3389/fmed.2026.1800406)
Supplement: Supplementary file 1 [file Supplementary_file_1.docx]

**Figure captions Supplementary Material**

**Figure S1.** Evaluation of *IL-1β* at different concentrations on cell viability **(a)** and apoptosis **(b).** Human osteoarthritic (OA) chondrocytes were incubated with interleukin *(IL)-1β* at different concentrations (2.5 ng/mL, 5 ng/mL, 10 ng/mL) alone or in the presence of Levico water (LW) at 50% of concentration for 24 h. The data analysis was calculated as fold change to control culture (CTRL = equal to 100 or 1). Data were represented as mean ± standard deviation. *p < 0.05, **p < 0.01 versus CTRL; °p < 0.05, °°p < 0.01 versus *IL-1β*.

**Figure S2**. Levico water (LW) effect on viability**.** Chondrocytes were incubated with LW at 50% **(a)** or 25% **(b)** for different time points, 0 h, 12 h, 24 h, 36 h, and 48 h. The data analysis was calculated as fold change control culture (CTRL = equal to 100). Data were represented as mean ± standard deviation.
