# Supplementary material for: A sulfate-arsenical-ferruginous water affects apoptosis, oxidative stress and the gene expression of inflammatory mediators and of a panel of MicroRNA in IL-1β stimulated human osteoarthritic chondrocytes
Source: Front Med (Lausanne). 2026 Apr 20;13:1800406. doi: 10.3389/fmed.2026.1800406 (PMC13137369; doi:10.3389/fmed.2026.1800406)
Supplement: Supplementary file 2 [file Table_1.docx]

| **Table S1.** Demographic and clinical characteristics of patients with hip osteoarthritis (OA) subjected to total arthroplasty | |  |
| --- | --- | --- |
| Age (years) (Mean ± SD) | **69.72 + 6.5** | |
| Gender |  | |
| Male (no) | **2** | |
| Female (no) | **3** | |
| BMI (kg/m²) (Mean ± SD) | **24.54 + 4.7** | |
| Disease duration (years) (Mean ± SD) | **12.1 + 6.4** | |
| Radiographic OA severity  (Kellgren- Lawrence score) IV (no) | **5** | |
| Comorbidities (no) |  | |
| Hypertension | **1** | |
| Diabetes | **0** | |
| Respiratory Diseases | **0** | |
| Gastroenteric Disorders | **2** | |
|  |  | |
|  |  | |
|  |  | |
| Concomitant treatments (no) |  | |
| Paracetamol | **5** | |
| Non steroidal antiinflammatory drugs (NSAIDs) | **0** | |
|  |  | |
|  |  | |
| Exercises | **2** | |
