# Supplementary material for: A sulfate-arsenical-ferruginous water affects apoptosis, oxidative stress and the gene expression of inflammatory mediators and of a panel of MicroRNA in IL-1β stimulated human osteoarthritic chondrocytes
Source: Front Med (Lausanne). 2026 Apr 20;13:1800406. doi: 10.3389/fmed.2026.1800406 (PMC13137369; doi:10.3389/fmed.2026.1800406)
Supplement: Supplementary file 3 [file Table_2.docx]

| **Table S2.** Chemical and physical properties of sulfate-arsenical-ferruginous Levico water (LW) provided by Levico Terme Health Resort (Levico Terme, Italy) used in the study. (Analysis by APPA, Agenzia Provinciale per la protezione dell’ambiente (Trento,Italy) (report 21LA02343). |
| --- |

| **Properties** | **Results** | **Unit of measure** |
| --- | --- | --- |
| Acidity (pH) | 1.9 | - |
| Temperature at source | 9.4 | °C |
| Conductivity | 10,400 | µS/cm |
| TDS | 6927 | mg/l |
| Carbon dioxide (CO₂) | 22 | mg /L |
| Cations |  | mg/L |
| Aluminium(AL) | 49.7 |  |
| Ammonium ion (NH₄^+^) | 0.46 |  |
| Antimony (SB) | 0.294 |  |
| Cadmium (Cd) | 0.221 |  |
| Calcium (Ca^++^) | 128.4 |  |
| Copper (Cu) | 16.9 |  |
| Iron (Fe) | 1550 |  |
| Iron(ferric) (Fe^+++^) | 175 |  |
| Iron (ferrous) (Fe^++)^ | 1375 |  |
| Lead (Pb) | 2.08 |  |
| Magnesium (Mg^++^) | 87.7 |  |
| Manganese (Mn) | 6.97 |  |
| Potassium (K^+^) | 2.3 |  |
| Sodium (Na^+^) | 2.2 |  |
| Strontium (Sr^++^) | 0.10 |  |
| Zinc (Zn) | 144 |  |
| Anions |  | mg/L |
| Arsenic (As) | 4.73 |  |
| Chloride (CL¯) | 1.2 |  |
| Fluoride (F¯) | 0,90 |  |
| Silica (SiO₂) | 26 |  |
| Sulphate(SO₄¯) | 4680 |  |
