# Supplementary material for: A sulfate-arsenical-ferruginous water affects apoptosis, oxidative stress and the gene expression of inflammatory mediators and of a panel of MicroRNA in IL-1β stimulated human osteoarthritic chondrocytes
Source: Front Med (Lausanne). 2026 Apr 20;13:1800406. doi: 10.3389/fmed.2026.1800406 (PMC13137369; doi:10.3389/fmed.2026.1800406)
Supplement: Supplementary file 4 [file Table_3.docx]

**Table S3.** List of primers used for quantitative real time PCR.

| **Genes** | **Cat. No. (Qiagen)** |
| --- | --- |
| *IL-1β* | QT00021385 |
| *IL-6* | QT00083720 |
| *TNF-α* | QT00029162 |
| *BCL2* | QT00000721 |
| *MMP-1* | QT00014581 |
| *MMP-13* | QT00001764 |
| *Col2a1* | QT00049518 |
| *ACAN* | QT00001365 |
| *SOD-2* | QT01008693 |
| *CAT* | QT00079674 |
| *NRF2* | QT00027384 |
| *NFKB1* | QT00063791 |
| *RELA* | QT01007370 |
| *CHUK* | QT00040614 |
| *ACTB* | QT00095431 |
| *miR-34a* | MS00003318 |
| *miR-140* | MS00003500 |
| *miR-146a* | MS00003535 |
| *miR-181a* | MS00006692 |
| *miR-let7e* | MS00031801 |
| *SNORD-25* | MS00014007 |

Abbreviations: IL-1β = interleukin 1β, IL-6 = interleukin 6, TNF-α = tumor necrosis factor α, BCL2 = B-cell lymphoma 2, MMP-1 = metalloproteinase 1, MMP-13 = metalloproteinase 13, Col2a1 = type II collagen, ACAN = aggrecan, SOD-2 = superoxide dismutase 2, CAT = catalase, NRF2 = nuclear factor erythroid 2 like 2, NFKB1 = Nuclear Factor Kappa B Subunit 1 (p50), RELA = pro-to-Oncogene, NF-KB Subunit (p65), CHUK = inhibitor of nuclear factor kappa-B kinase subunit alpha (IKK-α), ACTB = Actin Beta, miR = microRNA, SNORD-25 = Small Nucleolar RNA, C/D Box 25.
